# Headache disorders and the Global Burden of Disease study: deficiencies, challenges and opportunities for betterment

**DOI:** 10.1186/s10194-026-02490-0

**Published:** 2026-08-11

**Authors:** Andreas Kattem Husøy, Latifa Adarmouch, Amalie Berring-Uldum, Raquel Gil-Gouveia, Rigmor Jensen, Risako Shirane, Timothy J. Steiner

**Affiliations:** 1 NorHead, Department of Neuromedicine and Movement Science, Norwegian University of Science and Technology, Edvard Griegs, Gate, Trondheim, Norway; 2 Department of Neurology and Clinical Neurophysiology, St Olavs University Hospital, Trondheim, Norway; 3 Biosciences and Health Research Laboratory, School of Medicine and Pharmacy, Cadi Ayyad University, Marrakesh, Morocco; 4 Clinical Research Department, Mohammed VI University Hospital, Marrakesh, Morocco; 5 Department of Pediatrics and Adolescent Medicine, Copenhagen University Hospital – Herlev and Gentofte, Herlev, Denmark; 6 Hospital da Luz Headache Center, Neurology Department, Hospital da Luz, Lisbon, Portugal; 7 Center for Interdisciplinary Research in Health, Universidade Católica Portuguesa, Lisbon, Portugal; 8 Danish Headache Centre, Department of Neurology, Rigshospitalet Glostrup, Faculty of Health and Medical Sciences, University of Copenhagen, Copenhagen, Denmark; 9 Department of Neurology, Yale School of Medicine, New Haven, CT USA; 10 Department of Neurology, University of Copenhagen, Copenhagen, Denmark; 11 Department of Brain Sciences, Imperial College London, London, UK

**Keywords:** Headache disorders, Migraine, Tension-type headache, Medication-overuse headache, Health loss, Disability, Public health, Global burden of disease, Global campaign against headache

People with headache, and researchers, health-care providers, health-care policy makers and others with an interest in headache disorders, have all been very well served by the Global Burden of Disease study (GBD). From 2000 to 2023, GBD has generated constantly updated population estimates of headache-attributed ill health (expressed in years lived with disability [YLDs]) [1–8]. Each time, it has reaffirmed the magnitude of the global health loss, raising awareness—more than any other programme of activity—of the enormous negative impact of headache disorders on health (Table 1).

**Table 1:** GBD 2000–2023 and headache disorders

GBD iteration	Key features and messages
GBD 2000 [1]	the second iteration (after GBD 1990) conducted by WHO and the first to include migraine, ranking it 19th highest cause of YLDs worldwide, a revelation that led directly to WHO’s partnership in the Global Campaign against Headache [9]
GBD 2010 [2]	the first iteration under the stewardship of IHME, introducing entirely new methodology, the first to include TTH, and the first to include data from the Global Campaign against Headache, ranking migraine seventh highest cause of YLDs [10]
GBD 2013 [3]	the first to include MOH, and ranking migraine, TTH and MOH collectively as third highest cause of YLDs [11]
GBD 2015 [4]	ranking migraine third highest cause of YLDs in under 50s [12]
GBD 2016 [5]	the first to support specific analyses of headache disorders [13], ranking migraine second highest cause of YLDs, and top among young adults [14]
GBD 2019 [6]	highlighting the burden of migraine among young women (for whom it remained the top cause of YLDs) [15]
GBD 2021 [7]	the first with a focus on children (5–14 years), ranking headache disorders fourth highest cause of YLDs in this age group [16]
GBD 2023 [8]	the most recent iteration, and the second to support specific analyses of headache disorders [17], highlighting female health loss (more than twice that among males) and that one fifth of the health loss associated with headache is attributable to MOH [18]

GBD: Global Burden of Disease study; WHO: World Health Organization; YLDs: years lived with disability; IHME: Institute for Health Metrics and Evaluation; TTH: tension-type headache; MOH: medication-overuse headache

Yet, headache disorders remain only partially accounted for. Indeed, GBD 2023, supporting the first specific analyses of headache disorders since GBD 2016 [13], laid bare the many limitations of GBD with regard to them [17].

Calls to action on headache burden, fuelled by GBD findings, have followed each iteration over the past 15 years [10–12, 14–16, 18]. This editorial is not intended to be yet another: it is not addressed to policy makers. Its purposes, as the headache community increasingly recognizes the unique value of GBD, are to explain, with regard to headache, what GBD does and does not do, what it can and (currently) cannot do, and the opportunities that exist to contribute to its betterment. GBD methodology can doubtless be improved, and the Institute for Health Metrics and Evaluation (IHME) strives continuously to do this. But its resources are limited. If we want headache to be fully captured and accounted for in GBD, we need to help. Some of the means for betterment lie only with the headache community.

We hope, in highlighting these, to instil an enthusiasm for picking up the challenges.

## How GBD works for headache

GBD, and the data-acquisition and analytical methods it has developed during the last 20 years, have transformed population-health science by providing standardized measures of disease burden. Central to its framework is the disability-adjusted life year (DALY), a metric combining premature mortality (expressed as years of life lost [YLLs]) and lost health among the living (expressed as YLDs) [8].

The Global Campaign against Headache [9, 19] has contributed to all iterations of GBD from 2010 onwards, supporting population-based studies worldwide to fill data gaps [20, 21], feeding the data to GBD, and collaborating with IHME in the modelling and interpretation of headache data.

Only three headache types feature in GBD [17]: migraine (since GBD 2000 [1]), tension-type headache (TTH; since GBD 2010 [2]) and medication-overuse headache (MOH; since GBD 2013 [3]). There are more than 200 others identified in the International Classification of Headache Disorders (ICHD) [22], but these three account for almost all of the impact on population health. Migraine and TTH are separately modelled as “definite” or “probable” diagnoses (according to ICHD criteria [22]), with combined estimates then generated for each [17]. MOH-attributed burden is re-attributed to migraine or TTH, being in almost all cases a complication of one or the other, partitioned between them in the ratio 73.2:26.8 according to a meta-analysis of three published studies [17].

GBD attributes no premature mortality to these disorders, so headache-attributed burden is entirely captured by YLDs. Three measures contribute to YLD estimation: 1) prevalence; 2) time spent in the symptomatic state (*ie*, with headache); and 3) the disability weight (DW) assigned to the symptomatic state [17]. The formula is as follows:

$$\eqalign{{\bf{YLDs}} & = {\bf{prevalence}} \cr & \times {\bf{timeinsymptomaticstate}} \cr & \times {\bf{disabilityweight}} \cr} $$

For headache, estimates of prevalence and of time spent in the symptomatic state are derived solely from population-based epidemiological studies [17].

DWs were derived for GBD 2010 and refined for GBD 2013 [23]. Despite their name, they reflect diminished health, not disability [23]. They are, essentially, public perceptions, derived from a very large global reference panel [23], of the health impairments associated with the symptomatic states of each disorder. We say much more about these below.

## Deficiencies, challenges and opportunities for betterment

GBD 2010 introduced entirely new methodology, which, with each subsequent iteration, has undergone stepwise refinement. But major challenges—epidemiological (data-related), diagnostic, analytical and conceptual—continue to limit the precision with which headache-attributed burden is estimated. These are the deficiencies; they are also the opportunities for betterment.

### Headache types in GBD

Of the >200 other headache disorders in ICHD, the majority are secondary [22]; burdens arising from these are attributed by GBD to the underlying causal disorders. This is conceptually correct: throughout, GBD attributes burden to whatever is the root cause. Nevertheless, several primary headache disorders not included in GBD are highly detrimental to health and functioning at individual level: notably, cluster headache and other trigeminal autonomic cephalalgias. Their prevalences are low (<1/1,000 [24]), so their impacts on population health are small. For the same reason, reliable estimates with acceptable confidence intervals appear to require studies with very large population samples (>100,000) conducted in multiple and diverse geographical settings.

To include these disorders, novel methodologies are needed not only to establish prevalence but also to assign DWs. While these must reflect health losses associated with ictal states, crucially they must also recognise that health is significantly impaired interictally [25].

### Data gaps

Even for migraine and TTH, reliable data are missing for large areas and populations of the world, particularly in low- and middle-income countries [17].

The Global Campaign has addressed many of these gaps by focusing its epidemiological studies on these countries [20, 21]. GBD addresses them by using hierarchical statistical models that extrapolate from neighbouring regions and past years (“borrowing strength over time and space” [8]). It is a pragmatic approach and the best replacement for absent empirical data in the many countries that cannot support these resource-demanding studies.

### Case ascertainment

An uncertain proportion of people with headache have both migraine and TTH. Cross-sectional epidemiological enquiry basing diagnoses on symptoms recalled over the preceding months cannot reliably separate different headache types within participants’ descriptive accounts. Many studies, again pragmatically, assign only a single headache diagnosis per participant, usually, if not explicitly, of the subjectively most bothersome type [26]. This approach favours migraine, and very probably overlooks coexisting TTH.

It is easy to demand that future epidemiological studies should better account for multimorbidity (two or more headache types in one participant), but this would be a highly challenging and resource-consuming requirement. If the purpose of enquiry is needs assessment to inform policy (for whom should headache services provide?), there may be insufficient added benefit from knowing who with migraine also has TTH to justify the added cost of obtaining that knowledge.

### Time in symptomatic state

For headache disorders, this key factor in YLD estimation (see formula above) is derived as the product of headache frequency and mean duration recalled over past months by participants in population-based studies [17]. Recall is vulnerable to error [27], and attendant bias—especially among children. But to obtain such data prospectively would again be highly challenging, while any attempt to do so would probably invite other biases (interest-bias in particular).

Global Campaign studies have addressed this issue by comparing estimates based on recall with those based on headache yesterday (headache on the day preceding survey enquiry). These studies confirm the existence of recall bias, but suggest, perhaps surprisingly, that it is towards underestimation [28].

Meanwhile, incorporation of age- and gender-specific time-in-symptomatic-state estimates into GBD 2023 was a significant methodological advance [17], reflecting, in particular, that females with headache spend more time with headache than their male counterparts. However, current estimates remain neither geographically nor time specific. Thus, the same symptomatic-state proportions are applied across countries and years, despite likely differences in healthcare access and treatment availability, which would be expected to influence time in symptomatic state. Generating geographically and temporally resolved estimates of time in symptomatic state should be an important objective for future GBD iterations, but this would undoubtedly require more high-quality epidemiological data to make such estimates reliable.

### Disability weights

DWs are perhaps the most challenging issue in GBD. Conceptually, DWs standardize non-fatal health losses attributable to health states associated with disease. DWs are on a scale from 0 (full health) to 1 (health impairment equivalent to death) in accordance with public perceptions of these health states, which, for the purpose, were described in simplified lay terms [23] (Table 2). Whether these descriptions accurately—or adequately—capture the health impairment associated with the symptomatic state of each headache disorder is very questionable: when developed (for GBD 2010), they were much constrained: the word allowance was limited, and subjective terms were to be avoided. On the basis of these descriptions, GBD currently assigns a DW of 0.441 to migraine, about half this (0.223) to MOH, but only 0.037 to TTH [23] (Table 2).

**Table 2:** Lay descriptions and assigned disability weights for headache disorders in the Global burden of Disease study

Health state^1^	Lay description	Disability weight (95% CI)
Migraine	Has severe, throbbing head pain and nausea that cause great difficulty in daily activities and sometimes confine the person to bed. Moving around, light and noise make it worse.	0.441 (0.294–0.588)
Tension-type headache	Has a moderate headache that also affects the neck, which causes difficulty in daily activities.	0.037 (0.022–0.057)
Medication-overuse headache	Has daily headaches, felt as dull pain and often lasting all day, with poor sleep, nausea and fatigue. The person takes medicine for the headaches, which provides little relief but is needed to avoid having worse symptoms.	0.223 (0.146–0.313)

^1^ The symptomatic (ictal) state of each disorder

The almost 12-fold differential between migraine and TTH requires review: many people with TTH report throbbing pain, phonophobia and aggravation with physical activity—symptoms not represented in the lay description.

In 2010, and in 2013 when DWs were revised [23], GBD did not explicitly take account of definite and probable migraine: the same DWs were assigned to each (likewise for definite and probable TTH). This remains the case. In population-based studies, most diagnoses of probable rather than definite migraine (about 60% [29]) are due to durations outside the ICHD-mandated range of 4–72 hours [22]. Since duration is a factor in the calculation of time in symptomatic state, this is fully captured in YLD estimations. Since, also, such cases must meet all other criteria, the case for assigning a different DW in these instances is not strongly arguable. For the others – those who fail either criterion C (headache characteristics) or criterion D (accompanying symptoms) [22] – this is not so. It is from this minority (about 40% [29]) that a more compelling case for review arises.

There are now sufficient population-based data from countries worldwide in the Global Campaign’s dataset of *N* = 75,000 [30] to support a revision based on empirical evidence of health impairment and disability. But setting DWs according to how *affected* people report outcomes would require a major methodological shift in how GBD assigns values to health states.

### Children and adolescents

There is abundant evidence that headache episodes among young people are of relatively short duration (very often < 2 hours) [31–34]. However, current YLD estimates fail to account for this: in the absence of specific estimates of time in symptomatic state for children and adolescents, data on headache frequency and duration among young adults are used to fill this gap. The Global Campaign’s dataset [30] is equipped to provide a better solution.

A major additional uncertainty is how well the lay descriptions in Table 2, purporting to describe migraine, TTH and MOH in adults, apply to these disorders as they manifest among children and adolescents. Furthermore, in population-based studies, large proportions of children and adolescents have reported mild or moderate headache typically lasting < 1 hour, not fulfilling ICHD criteria for (definite) migraine or TTH. For this, the term *undifferentiated headache* (UdH) has been coined, supposing it to be an expression by the immature brain of a headache disorder that will evolve into one or the other [35]. UdH is not captured at all by GBD. While evidence of its prevalence is being gathered in Global Campaign studies around the world [31–34], and of its symptomatology, UdH lacks a DW.

### Extra-ictal burden

There is no recognition in GBD that health loss from headache disorders is attributable to anything other than the symptomatic state (the “headache phase”, in the case of migraine). Health impairment *between* attacks (interictal burden) matters, because it is potentially present for much longer periods than the attacks themselves. It has two principal contributors: anxiety in anticipation of the next attack, and avoidance behaviour to avert it, which leads to life-style compromise. Both have been shown in Global Campaign studies to add appreciably to disease burden [36]. Persisting symptoms, such as nausea or photophobia for migraine, can also cause functional impairment in the extra-ictal phase.

Expanding burden estimation in GBD to include extra-ictal burdens requires considerable and innovative adjustment to its quantifying methodology for headache disorders. But, for migraine at least, doing so would align GBD’s modelling more closely with our understanding of it as a complex neurological disorder.

### Risk factors

For several non-communicable diseases, GBD models incorporate known causal risk factors to strengthen prevalence predictions across populations and regions. Headache disorders are modelled without such inputs. This reflects an evidence gap rather than a lack of plausible candidates: socioeconomic status, sleep disturbance, obesity, psychological distress and variables related to lifestyle have all been associated with headache [37–40]. But the inability to integrate risk factors limits both the explanatory power and predictive capacity of current headache models, particularly in regions with sparse data.

Incorporation of risk factors into GBD requires robust evidence of causality, consistency across populations and quantified exposure–response relationships [41]. For headache disorders, this evidence remains lacking: longitudinal studies are needed, but these, again, are challenging.

## Looking forward

Headache disorders are a clear example of how non-fatal diseases can impose enormous population-level ill-health burdens, a reality that GBD, despite its deficiencies, has been instrumental in bringing sharply into focus.

The relationship between the headache community and the GBD enterprise from GBD 2010 onwards has been symbiotic, and fundamentally productive. Headache epidemiological research has matured from poorly developed infancy into a respectable science [26, 42], generating a large volume of microdata filling many of the large data gaps in low- and middle-income countries and among children and adolescents [21, 30]. GBD has put meaning to these data, providing headache researchers and advocates with a unique and powerful framework for communicating disease burden to policymakers, funding agencies and other stakeholders.

The next iteration is likely to be GBD 2025. For headache it will be an important revision, including a wealth of new data gathered since GBD 2017. There will be further modelling refinements to take account of at least some of the deficiencies identified here, every one of which creates an opportunity for betterment—more comprehensive and more nuanced estimates—although the processes of improvement will extend well beyond GBD 2025.

Improvement is not merely an academic ambition. GBD rankings are used by the World Health Organization, national health ministries and funding bodies. High positions among DALY or YLD rankings shape decisions to include disorders in national health strategies, and to allocate health and research resources to them. Underestimation of headache-attributed burden has a cost: fewer of these resources allocated than headache disorders merit.

## Data Availability

Not applicable.

## Abbreviations

DALY: Disability-adjusted life year
DW: Disability weight
GBD: Global Burden of Disease (study)
HARDSHIP: Headache-attributed restriction, disability, social handicap and impaired participation (questionnaire)
HY: Headache yesterday
ICHD: International Classification of Headache Disorders
IHME: Institute for Health Metrics and Evaluation
MOH: Medication-overuse headache
TTH: Tension-type headache
UdH: Undifferentiated headache
WHO: World Health Organization
YLD: Year lived with disability
YLL: Year of life lost
